# Stumped by Appendicitis: A Rare Cause of Acute Abdominal Pain

**DOI:** 10.7759/cureus.50557

**Published:** 2023-12-15

**Authors:** Nada A Mohammed, Mustak Dukandar

**Affiliations:** 1 Emergency Department, Tawam Hospital, Al Ain, ARE

**Keywords:** lower right quadrant pain, computed tomography, appendectomy, emergency department, stump appendicitis

## Abstract

Acute appendicitis is one of the most common diagnoses in the emergency department. As with other surgical procedures, post-appendectomy complications are numerous and can be either immediate or delayed. Stump appendicitis is an underreported and underrecognized complication that is often diagnosed radiologically while ruling out other diagnoses.

We report a case of a 26-year-old male presenting with acute right lower quadrant abdominal pain. Although he initially denied any surgical history, a focused abdominal exam revealed an incisional scar which turned out to be the result of an appendectomy nine years ago. The patient was worked up for alternate causes of right lower quadrant pain.

Investigations revealed high inflammatory markers and hematuria. We proceeded with a non-contrast CT scan to rule out vesicoureteric junction stone. Instead, the scan was suggestive of stump appendicitis. The patient was admitted and treated conservatively.

Maintaining a high index of suspicion for stump appendicitis, especially in patients with a clinical picture typical of appendicitis but a history of appendectomy, is key to making an early diagnosis and avoiding further complications.

## Introduction

Acute appendicitis is one of the most prevalent causes of presentation to the emergency department (ED) [1]. With the treatment being resection, whether open or laparoscopic, it is not uncommon for patients to present with complications of appendectomy. One of the delayed complications of this procedure is stump appendicitis, a once rare entity with a now increasing incidence [2]. The estimated figure in literature is one in 50,000, although this is likely underestimated and the condition underreported [3].

Stump appendicitis can occur as soon as two months and as late as 50 years post-appendectomy [4]. It is often diagnosed radiologically on an abdominal CT scan performed to rule out other diagnoses [5]. A lack of awareness by the ED physician can lead to delayed diagnosis, which inadvertently leads to delayed treatment and an increased complication rate [6]. We report a case of a young male presenting with acute abdominal pain and a history of appendectomy. Stump appendicitis was diagnosed incidentally on CT and the patient was managed conservatively.

## Case presentation

A 26-year-old male presented to ED with a one-day history of right lower abdominal pain associated with nausea and one episode of vomiting. The pain was of gradual onset, dull achy character, mild to moderate intensity, and intermittent course. He denied fever, altered bowel habit, or dysuria; review of systems was otherwise unremarkable. During the initial interview, he denied any past medical or surgical history. His only medications were simple analgesics, he was a non-smoker and did not consume alcohol, and family history was non-contributory. 

Vitally, the patient was stable and afebrile. Focused abdominal examination revealed an incisional appendectomy scar; when asked about this finding, the patient then added that he underwent an open appendectomy nine years ago. He also had moderate right lower quadrant (RLQ) tenderness with rebound and mild guarding. No other peritoneal signs were present, and the systemic examination was otherwise normal. At this point, appendicitis was ruled out and the work-up was aimed at identifying alternate causes for his RLQ pain.

Investigations

**Figure 1:**
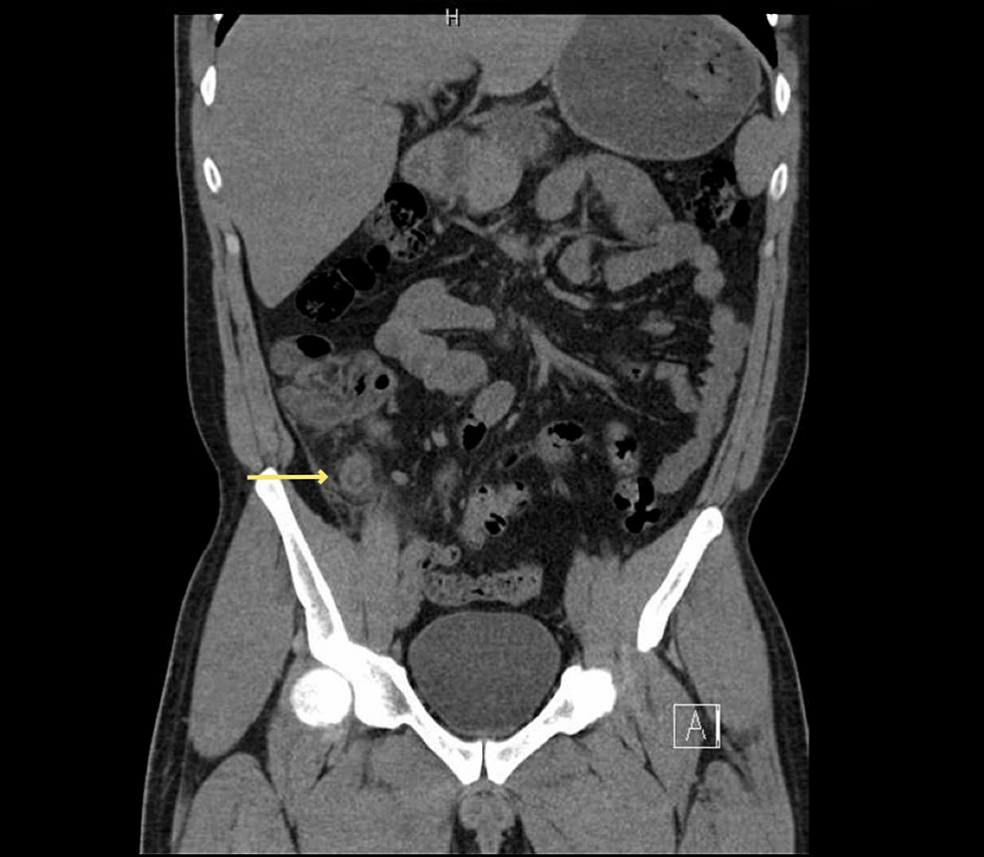
Non-contrast abdominal CT image showing wall thickening of a blind-ended tubular structure arising from the cecal pole (yellow arrow), suggestive of inflamed appendiceal stump.

Laboratory investigations showed mild leukocytosis (12.6 x 109/L) with neutrophilia (8.6 x 109/L) and an elevated C-reactive protein (CRP) (140.4mg/L). Urinalysis was positive for erythrocytes. The high inflammatory markers combined with hematuria led to the consideration of vesicoureteric junction stone as a possible diagnosis; thus, we proceeded to a non-contrast CT scan of the abdomen and pelvis, which revealed diffuse wall thickening of a blind-ended, tubular structure arising from the cecal pole, suggestive of inflamed appendicular stump (Figure 1). A repeat CT with contrast further delineated the inflammatory changes to include the cecum and proximal ascending colon with surrounding fat stranding and multiple prominent reactive lymph nodes. 

Differential diagnosis

Although the clinical picture of RLQ pain and tenderness allows for a wide range of differential diagnoses, it is confounded by the history of appendectomy. One literature review concluded that the most common misdiagnoses were constipation, gastroenteritis and right-sided diverticulitis [7]. 

Treatment

General surgery was consulted, and no acute surgical intervention was deemed necessary. The patient was then admitted as a case of stump appendicitis and managed conservatively. He was kept nothing by mouth (NPO) and started on intravenous analgesia, anti-emetics and antibiotics (cefuroxime, metronidazole). During admission, the gastroenterology service was consulted and the patient underwent colonoscopy which was grossly normal. Random biopsies were taken for pathology examination. 

Outcome and follow-up

The patient was discharged three days later on an oral course of the same antibiotics for a total of seven days. On a follow-up appointment with gastroenterology, he reported improved symptoms and pathology reports showed no significant histological abnormality. Repeat labs showed a downward trend of inflammatory markers. The final diagnosis was stump appendicitis.

## Discussion

Stump appendicitis (SA) is defined as interval inflammation of residual appendicular tissue after appendectomy [5]. First described in 1945 by Rose, its incidence has since been slowly rising [2]. This has been attributed to laparoscopic surgery by some authors but refuted by others; an alternative explanation is the increasing awareness to this clinical entity in recent years [8]. However, a low index of suspicion remains one of the principal reasons behind delayed diagnosis and treatment [9]. A 2020 literature review by Enzerra et al. counted 160 cases reported in published literature; another review in the same year by Burbano et al. concluded that “the incidence of stump appendicitis seems to be higher than the one reported of 1 in 50,000” [10,11]. 

Several anatomic and surgical risk factors have been suggested to increase the risk of SA; the most consistent seem to be inappropriate identification of the base of the appendix at the time of surgery and length of the residual stump [4]. Although there is no clinical consensus on the exact measurement, the general recommendation is that the stump be less than 5 mm long [12]. A disputed risk factor is undergoing laparoscopic as opposed to open appendectomy. Theoretically, “the lack of a three-dimensional approach and the absence of a tactile return” increase the risk of SA post-laparoscopic appendectomy by increasing the length of tissue left behind [2]. Interestingly, one review found the incidence post-laparoscopy to be less than half of that post open appendectomy [13]. Ultimately, surgical technique and operator experience play a role in the complication rate of any procedure. Other risk factors include a retro-colic position of the appendix, poor blood supply, and appendicolith formation [5]. 

Clinical presentation of SA is similar to that of acute appendicitis, further confounding the diagnostic process. Although no one symptom or sign is specific, the most common findings are RLQ pain, leukocytosis, peritonism, fever, nausea and vomiting [11]. Epidemiologically, the diagnosis is made in patients ranging from eight to 80 years, with a median age of 33 years, and a male-to-female ratio of 1.1:1 [14]. Abdominal imaging is often needed to confirm the diagnosis, with computerized tomography (CT) being the gold standard [15]. Compared to ultrasound scans, CT is more sensitive and specific and can simultaneously exclude other abdominopelvic disease entities [8]. Findings are similar to those of acute appendicitis and can include cecal wall thickening, free fluid, abdominal collections or abscesses- as well as identification of the appendicular stump [8]. Less frequently, barium enema and colonoscopy can aid in the diagnosis [16]. In cases of ambiguity or diagnostic uncertainty, a decision to proceed to diagnostic laparoscopy can be made [17]. 

The vast majority of reported cases have cited a completion appendectomy as the treatment of choice for SA, with the open approach being preferred to laparoscopic surgery [6]. This may be due to the fact that a delayed diagnosis leads to more complicated presentations, such as stump gangrene, perforation, and peritonitis [5]. One review cited a perforation rate as high as 70% [14]. Conservative management with parenteral antibiotics and analgesia with or without colonoscopy may be appropriate for some patients, such as the case reported above; although it is less commonly described in the literature [4]. Potential uses of colonoscopy are washout, pus drainage and clearance, and appendicolith removal with a snare [8]. An interval appendectomy has also been suggested by some authors after the acute inflammatory phase subsides with medical management to improve intra-operative visualization of the stump and prevent recurrence [4]. 

## Conclusions

Right lower quadrant abdominal pain comes with a wide range of differential diagnoses. In patients with a clinical picture of acute appendicitis and history of appendectomy, it is the emergency physician’s responsibility to maintain a high index of suspicion for stump appendicitis. The gold standard for diagnosis is an abdominal CT. Prompt identification and treatment- primarily with a completion appendectomy- is key to avoid complications such as perforation.
